# The Hygiene of the Sick-Room

**Published:** 1893-01

**Authors:** 


THE HYGIENE OF THE SICK-ROOM. A book for nurses and others, being a brief consideration of Asepsis, Antisepsis, Disinfection, Bacteriology, Immunity, Heating, and Ventilation, and kindred subjects, for the use of nurses and other intelligent women. By WILLIAM BUCKINGHAM CANFIELD, A. M., M. D., Lecturer on Clinical Medicine and Chief of Chest Clinic, University of Maryland; Visiting Physician to Bay View Hospital; Visiting Physician to the Union Protestant Infirmary, etc., Baltimore. Philadelphia: P. Blakiston, Son & Co., 1012 Walnut street. 1892. Cloth; price, $1.50.

This little book is entitled by its author, "A Book for Nurses and *others*." The "others," in our opinion, should include all mothers and all medical students. After the introduction, which contains some valuable general advice to nurses, and tells why they should have a knowledge of the causes of contagion and the modes of infection, the work proper begins with a chapter on Bacteriology, which gives enough of the subject to impress the mind of the nurse or the mother with the great importance of the subject without fatiguing her with, to her, incomprehensible details.

The second chapter, on Infection and Disinfection, is the one that makes the book of value to the medical student. We know of no other book that treats this subject so briefly and yet so well. In the altogether too limited time that is devoted to medical education, the student cannot spare the amount necessary to carefully study a long treatise on these subjects, and this chapter contains the essentials. The next thirteen chapters are devoted to the consideration of the infectious diseases, one chapter being allotted to each disease. The chapters devoted to the consideration of Tuberculosis and Typhoid Fever are particularly good. The chapter on The Bacteria of Surgical Diseases shows excellently the necessity for "surgical cleanliness," and the difference between surgical cleanliness and æsthetic cleanliness. Then follows an excellent chapter on The Care of the Mouth. If nurses would "read, learn, mark, and inwardly digest" this chapter, many a patient would be anxious for food earlier in his convalescence, and would be better able to digest it when given. The chapter on Ophthalmia Neonatorium is one that every obstetric nurse should learn by heart.

The chapter on Ventilation and Heating is very good, embodying the results of the best thought in sanitary science on that subject.

Then follows a chapter on Immunity and Protection from Disease, which gives very kindly the several theories on that subject.

The book ends with a chapter on Food, which contains a number of important facts in regard to foods and their digestibility, and the best ways of administering them. It does not pretend in any way to be complete. The few hints here given may help the nurse in the right direction, and she can fill up the gaps by study in physiology, cooking, and other branches. This excellent little work is made complete by a full alphabetical index. We gladly recommend it as a text-book in all Training Schools for Nurses. The binding and letter-press leave nothing to be desired.

DeL. R.

